# Infection of Type I Interferon Receptor-Deficient Mice with Various Old World Arenaviruses: A Model for Studying Virulence and Host Species Barriers

**DOI:** 10.1371/journal.pone.0072290

**Published:** 2013-08-22

**Authors:** Toni Rieger, Doron Merkler, Stephan Günther

**Affiliations:** 1 Department of Virology, Bernhard-Nocht-Institute for Tropical Medicine, Hamburg, Germany; 2 German Centre for Infection Research (DZIF), partner site Hamburg, Germany; 3 Department of Neuropathology, Georg-August-University, Göttingen, Germany; 4 Division of Clinical Pathology, Geneva University Hospital and Department of Pathology and Immunology, Geneva, Switzerland; Tulane School of Public Health and Tropical Medicine, United States of America

## Abstract

Lassa virus causes hemorrhagic Lassa fever in humans, while the related Old World arenaviruses Mopeia, Morogoro, and Mobala are supposedly apathogenic to humans and cause only inapparent infection in non-human primates. Here, we studied whether the virulence of Old World arenaviruses in humans and non-human primates is reflected in type I interferon receptor deficient (IFNAR^-/-^) mice by testing several strains of Lassa virus vs. the apathogenic viruses Mopeia, Morogoro, and Mobala. All Lassa virus strains tested—Josiah, AV, BA366, and Nig04-10—replicated to high titers in blood, lung, kidney, heart, spleen, brain, and liver and caused disease as evidenced by weight loss and elevation of aspartate and alanine aminotransferase (AST and ALT) levels with a high AST/ALT ratio. Lassa fever-like pathology included acute hepatitis, interstitial pneumonia, and pronounced disturbance of splenic cytoarchitecture. Infiltrations of activated monocytes/macrophages expressing inducible nitric oxide synthase and T cells were found in liver and lung. In contrast, Mopeia, Morogoro, and Mobala virus replicated poorly in the animals and acute inflammatory alterations were not noted. Depletion of CD4^+^ and CD8^+^ T cells strongly enhanced susceptibility of IFNAR^-/-^ mice to the apathogenic viruses. In conclusion, the virulence of Old World arenaviruses in IFNAR^-/-^ mice correlates with their virulence in humans and non-human primates. In addition to the type I interferon system, T cells seem to regulate whether or not an arenavirus can productively infect non-host rodent species. The observation that Lassa virus overcomes the species barrier without artificial depletion of T cells suggests it is able to impair T cell functionality in a way that corresponds to depletion.

## Introduction

*Arenaviridae* are segmented negative strand RNA viruses. Their natural hosts are rodents of the superfamily *Muroidea*. Arenaviruses may cause hemorrhagic fever upon transmission to humans, though most members of the family are not associated with human disease. Africa is home to various Old World arenaviruses. Lassa virus is the most relevant pathogenic arenavirus in Africa [1]. It causes hemorrhagic Lassa fever in the West African countries of Nigeria, Liberia, Sierra Leone, Mali, and Guinea [2–4]. The case fatality rate in hospital is about 30%, but may be as high as 65% during nosocomial outbreaks [5,6]. Mopeia, Morogoro, and Mobala virus circulate in Mozambique/Zimbabwe, Tanzania, and Central African Republic, respectively [7–10]. They are supposedly apathogenic or low pathogenic to humans, as severe infections in patients have not been observed in the areas endemic for these viruses.

Lassa, Mopeia, and Morogoro virus share the natural host *Mastomys natalensis*, a rodent species prevalent in Sub-Saharan Africa [7,9–12]. The natural host of Mobala virus is *Praomys* sp. [8]. The strict association of a virus species with its host species indicates that arenaviruses may not easily establish a productive infection in non-host rodents or other mammals. However, virus–host co-phylogenies suggest that African arenaviruses have switched their hosts during evolution [13]. The species barriers to be hurdled by an arenavirus to infect a non-host species are not defined.

The pathogenesis of Lassa fever is still poorly understood. Several factors may be involved: immunosuppression due to impairment of dendritic cell activation and T cell response [14–16], activation of macrophages [17,18], and disturbance of coagulation and cytokine networks [19–21]. The pathophysiological cascade eventually leads to organ failure, in particular of the kidney, shock, and encephalopathy [6,19,22]. Virus can be isolated from virtually all organs [23]. Classical biochemical marker of the infection is an elevated aspartate aminotransferase (AST) level in serum, which is higher than the alanine aminotransferase (ALT) level [18,19,23]. Pathological lesions include hepatocellular necrosis with macrophage activation and minimal lymphocyte infiltration, splenic necrosis, renal tubular injury, interstitial nephritis, interstitial pneumonia, and myocarditis [18,23,24]. Similar changes are seen in experimental Lassa fever models, namely Lassa virus-infected non-human primates and inbred guinea pigs [20,25–32]. Mopeia and Mobala virus are benign in both models [26,31,33,34], while data on Morogoro virus are not yet available.

We and others have recently shown that mice, which are naturally resistant to Lassa virus infection, may be rendered susceptible to infection by knockout of the interferon alpha/beta receptor (IFNAR^-/-^ mice) and therefore may represent a suitable model to study Lassa virus infection [35,36]. Marked Lassa virus strain-specific differences in susceptibility were previously observed in a small animal model for Lassa fever based on HLA-A2 transgenic mice [17]. Therefore, we tested here the susceptibility range of IFNAR^-/-^ mice for various Lassa virus strains. In addition, we tested whether the apathogenic viruses Mopeia, Morogoro, and Mobala are able to productively infect mice devoid of a functional type I interferon system. The data obtained from these experiments suggest that IFNAR^-/-^ mice reproduce relevant aspects of arenavirus virulence in humans and non-human primates and provide new insights into the barriers preventing infection of non-host species by arenaviruses.

## Materials and Methods

### Ethics statement

This study was carried out in strict accordance with the recommendations of the German Society for Laboratory Animal Science under supervision of a veterinarian. The protocol was approved by the Committee on the Ethics of Animal Experiments of the City of Hamburg (Permit no. 52/10). All efforts were made to minimize the number of animals used for the experiments and suffering of the animals during the experiments. All staff carrying out animal experiments has passed an education and training program according to category B or C of the Federation of European Laboratory Animal Science Associations. The animal experiments in this study are reported according to the ARRIVE guidelines [37]. A total of 78 mice were used for this study and all mice were included in the analysis.

### Viruses

Lassa virus strains AV [38] and Nig04-10 [39], and Morogoro virus strain 3017/2004 [9] had been isolated in our laboratory and passaged ≤3 times before they have been used in this study. Lassa virus strains Josiah [40] and BA366 [12], Mopeia virus strain AN21366 [7], and Mobala virus strain 3099 [8] had been obtained from other laboratories with an unknown passage history. They have been passaged ≤3 times in our laboratory before use in this study. The virus stocks for inoculation of the animals were grown on BHK-21 cells, quantified by immunofocus assay (see below), and stored at -70°C until use.

### Mouse experiments

IFNAR^-/-^ mice (129Sv background) [41] were bred in the Specific Pathogen Free animal facility of the Bernhard-Nocht-Institute. Eight- to twelve-week-old female animals (weight median 19.8 g, range 14.8–25.6 g) were used for all experiments. A group size of 3–5 animals was expected to provide sufficiently accurate estimates of survival rate as well as mean and variance for viremia and clinical chemistry parameters. Experimental groups were age-matched. Animals of a group were kept together in a conventional cage without enrichments. They had ad libitum access to food and water. Experiments involving both Lassa virus and less pathogenic viruses were performed in the animal facility of the biosafety level (BSL) 4 laboratory to prevent a bias due to different housing conditions. The CD8^+^ and/or CD4^+^ T cell depletion experiments, which did not include Lassa virus, were conducted in the BSL-2 animal facility. Both BSL-4 and BSL-2 animal rooms had artificial light/dark cycles.

Three or five animals per group for experiments without or with organ collection, respectively, were infected by intravenous (i.v.) injection in the tail vein with 10^3^ or 10^5^ focus forming units (FFU) of virus in 200 µl minimal essential medium (MEM) (PAA Laboratories) containing 2% fetal calf serum (FCS). The mode of administration was chosen to facilitate comparability with our previous mouse model [17]. After infection, mice were monitored daily for body weight and signs of disease. Animals with severe signs of disease such as seizures, bleeding, abdominal distention, diarrhea, agony, or weight loss of >15% within 2 days shall be euthanized according to the protocol. However, no animal had to be euthanized due to these reasons in this study. Blood samples of 30–50 µl per animal were drawn by tail vein puncture in intervals of 3–7 days over a period of 21–37 days (6–7 blood drawings in total, respectively) for clinical chemistry and viremia measurement. For organ collection and at the end of the experiment, animals were euthanized with an isoflurane overdose followed by cervical dislocation. Organs were collected at day 9 or 10 post infection (p.i.) from 2 animals that have been randomly chosen from experimental groups with 5 animals, and analyzed for infectious virus titer and histopathological changes. CD8^+^ and/or CD4^+^ T cell populations were depleted by intraperitoneal administration of monoclonal antibodies YTS169 (anti-CD8) and YTS191 (anti-CD4) (Bio X Cell) on day -3 and day -1 of infection (300 µg of anti-CD8 and/or 300 µg of anti-CD4 per day). The efficiency of depletion was verified by flow cytometry and was >99%. Experiments were not replicated.

### Virus titration

Infectious virus particles in blood and organ samples were determined by immunofocus assay. Organ samples were homogenized in 500 µl MEM–2% FCS using Lysing Matrix D (MP Biomedicals) in a beat mill. Vero cells in 24-well plates were inoculated with 200 µl of serial 10-fold dilutions of sample. The inoculum was removed after 1 h and replaced by a 1%-methylcellulose medium overlay. After 5 days of incubation, cells were fixed with 4% formaldehyde, washed with phosphate-buffered saline (PBS), and permeabilized with 0.5% Triton X-100 in PBS. After washing with PBS and blocking with 10% FCS in PBS, cell foci infected with Lassa virus were detected with Lassa virus nucleoprotein (NP)-specific monoclonal antibody L2F1 [42]. Cell foci infected with Mopeia, Morogoro, or Mobala virus were detected with Old World arenavirus NP-specific monoclonal antibody L2D9 [42]. After washing, cells were incubated with peroxidase-labeled anti-mouse IgG. Foci were visualized with tetramethylbenzidine and counted.

### Clinical chemistry

Serum samples were diluted 1:10 in isotonic NaCl solution and analyzed for AST and ALT activity by using commercially available colorimetric assay kits at 25°C (detection limit for undiluted serum is 2.25 U/l for AST and 2.65 U/l for ALT) (Reflotron, Roche Diagnostics). Blood of 3 animals was pooled for clinical chemistry analysis of CD8^+^ and/or CD4^+^ T cell-depleted mice; otherwise parameters were measured for individual animals.

### Histology

Lung, kidney, heart, spleen, brain, and liver were collected, fixed in 4% formaldehyde in PBS, and embedded in paraffin. Sections were stained with hematoxylin/eosin (H/E) or processed for immunohistochemistry as follows: Upon inactivation of endogenous peroxidase with 3% hydrogen peroxide in PBS and blocking with 10% FCS in PBS, sections were incubated with rat anti-human CD3 (Serotec), which is crossreactive with murine CD3 on mouse T cells, rabbit anti-Iba-1 for monocytes/macrophages (Wako Pure Chemical Industries), anti-inducible nitric oxide synthase (iNOS), or rat anti-Lassa virus NP serum [17]. Primary antibodies were detected with biotinylated rat-specific (DakoCytomation) or rabbit-specific (Amersham) secondary antibody, followed by incubation with ExtrAvidin peroxidase (Sigma Aldrich). Peroxidase was visualized by using 3,3’-diaminobenzidine (Sigma Aldrich) as chromogen. Haemalaun was used for counterstaining of nuclei.

## Results

### Infection of IFNAR^-/-^ mice with various Lassa virus strains

**Figure 1:**
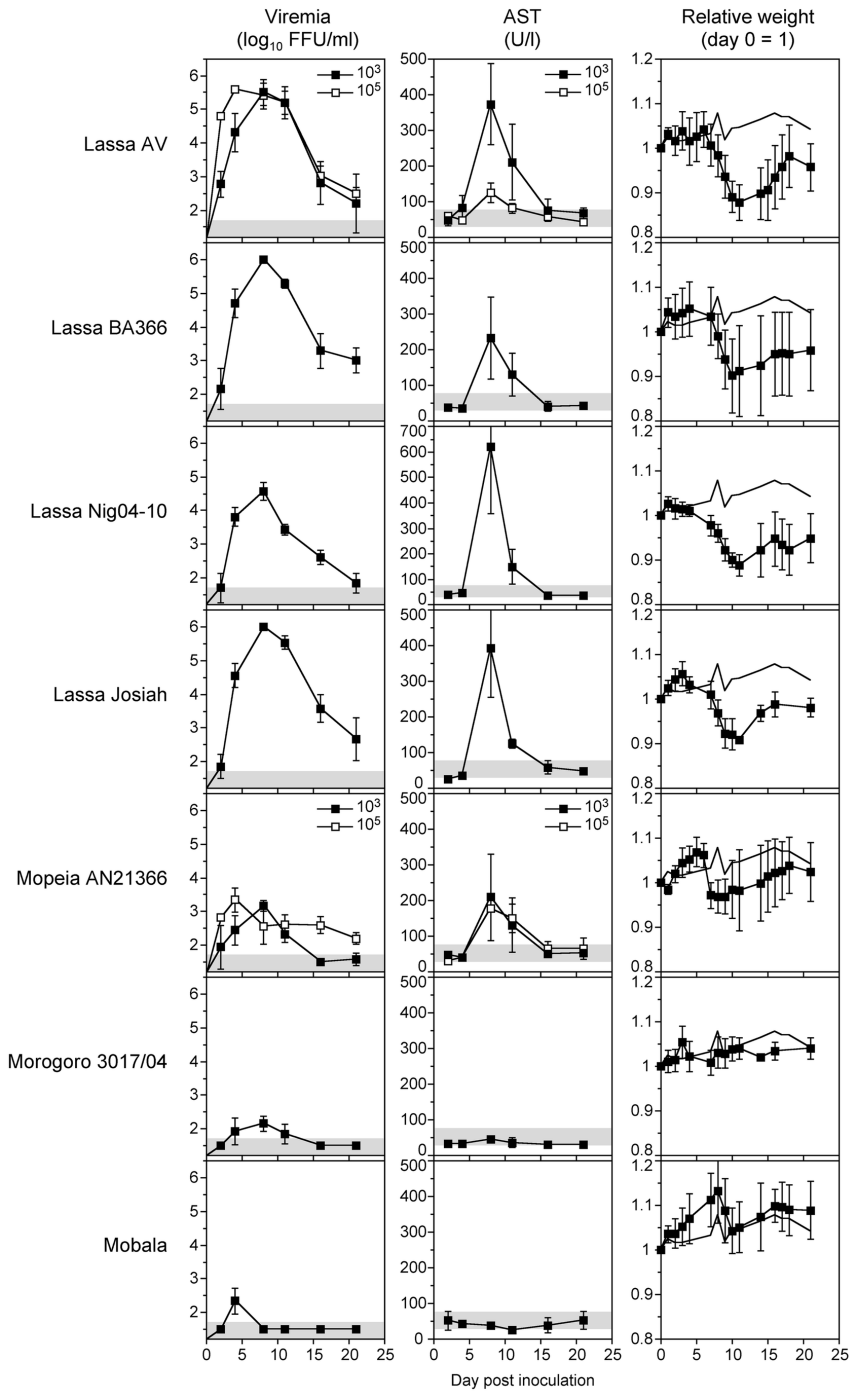
Viremia, AST, and body weight of IFNAR^-/-^ mice infected with various Old World arenaviruses. Groups of five animals were inoculated i.v. with 10^3^ FFU (filled squares) or 10^5^ FFU (open squares). Two of them were randomly euthanized at days 9–10 to collect organs. Mean and standard deviation are shown (n ≥ 3). The range of viremia below the detection limit of the immunofocusassay as well as the normal reference range of AST in mice [43] are shaded in grey. The weight curve of the uninfected controls is shown without data points.

In the first set of experiments, IFNAR^-/-^ mice were infected with various Lassa virus strains of different origin. Strains AV, Nig04-10, and Josiah had been isolated from humans with Lassa fever from Côte d’Ivoire, Nigeria, and Sierra Leone, respectively, while strain BA366 had been isolated from *M. natalensis* from Guinea [12,38–40]. Groups of 3 to 5 mice were inoculated with Lassa virus via the i.v. route and the level of viremia as well as the disease markers AST and body weight were measured at six time points up to day 21 after inoculation. AST is a classical biochemical marker of human Lassa fever [18,19,23]. In a pilot experiment with Lassa virus AV, low and high inoculation doses (10^3^ and 10^5^ FFU) were tested. Both doses resulted in an acute infection with up to 5.5 log_10_ FFU/ml of blood, although the peak of viremia was reached earlier with the higher inoculation dose (Figure 1). The elevation of AST was higher after inoculation with the lower dose. Therefore, all other experiments were performed with a dose of 10^3^ FFU per animal. IFNAR^-/-^ mice were susceptible to infection with all Lassa virus strains. The peak of viremia was around day 8 p.i. and ranged from 4.5 to 6 log_10_ FFU/ml blood (Figure 1). At day 21, the virus was not yet fully cleared with levels of viremia ranging from 2 to 3 log_10_ units FFU/ml blood. AST elevations were observed in all groups. AST peaks were seen at day 8 p.i. with values ranging from 250 to 650 U/l blood (Figure 1). ALT values were measured for BA366, Nig04-10, and Josiah and peaked at day 8 p.i. with 91 U/l (AST 232 U/l), 163 U/l (AST 620 U/l), and 133 U/l (AST 392 U/l), respectively (the normal reference range for 6 to 20-week old mice is 30–80 U/l for AST and 25–60 U/l for ALT [43]). Thus, like in human Lassa fever the values for AST were higher than for ALT [18,19,23]. All Lassa virus-infected animals lost about 15% of body weight by day 8 p.i. compared to non-infected controls (Figure 1). These data demonstrate that IFNAR^-/-^ mice are susceptible to various Lassa virus strains. Lassa virus infection causes non-lethal acute disease with elevation of AST and ALT and loss of weight.

### Infection of IFNAR^-/-^ mice with Mopeia, Morogoro, and Mobala virus

In a second set of experiments, we tested whether IFNAR^-/-^ mice are susceptible to Lassa virus-related arenaviruses that apparently do not cause disease in humans or other animal models [26,31,33,34]. Mice were inoculated with 10^3^ FFU of Mopeia, Morogoro, or Mobala virus. Viremia was significantly lower and of shorter duration compared to Lassa virus (Figure 1). Mopeia virus showed the highest peak level with about 3.5 log_10_ FFU/ml at day 8 p.i. The viremia of Morogoro and Mobala virus hardly reached 2.5 log_10_ FFU/ml. AST elevation with a maximum of 200 U/l blood was observed only for Mopeia virus, while the AST remained in the normal range after infection with Morogoro and Mobala virus (Figure 1). Similarly, a minor weight loss of about 5% compared to non-infected controls was seen for Mopeia virus, while Morogoro and Mobala virus-infected animals did not lose weight. Increasing the inoculation dose to 10^5^ FFU of Mopeia virus increased neither viremia nor AST values (Figure 1). Taken together, apathogenic arenaviruses are low pathogenic in IFNAR^-/-^ mice or cause inapparent infection.

### Virus titer in organs of IFNAR^-/-^ mice

**Figure 2:**
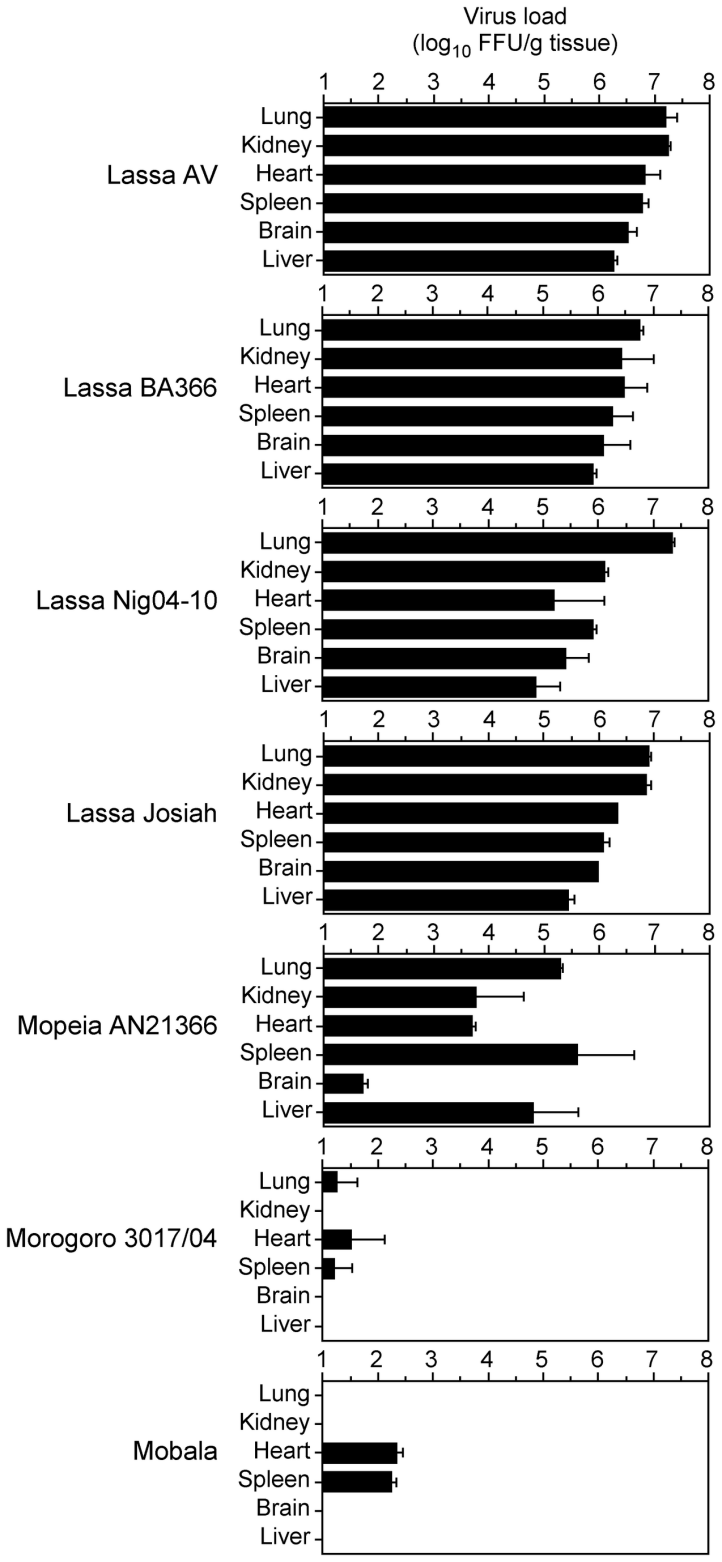
Virus titers in organs of IFNAR^-/-^ mice. Animals were inoculated i.v. with 10^3^ FFU of the indicated viruses and euthanized at day 9–10 p.i. to collect the organs. Mean and range are shown (n = 2).

Lassa virus is pantropic in humans and other animal models [23,25]. To determine the organ tropism of Lassa, Mopeia, Morogoro, and Mobala virus in IFNAR^-/-^ mice, animals were sacrificed 9 or 10 days p.i. and the virus titer in lung, kidney, heart, spleen, brain, and liver was determined. Lassa virus was detected in all organs with titers ranging from 5 to 7.5 log_10_ FFU/g tissue (Figure 2). The highest titer was found in lung with about 7 log_10_ FFU/g tissue. The organ titers for Mopeia, Morogoro, and Mobala virus were generally lower than for Lassa virus. In agreement with the data on viremia, Mopeia virus was found in all organs with titers ranging from 2 to 5.5 log_10_ FFU/g tissue, while Morogoro and Mobala virus were found only in some organs with titers lower than 2.5 log_10_ FFU/g tissue (Figure 2). The organ distribution of Mopeia virus differed from that of Lassa virus. In particular, the Mopeia virus titer in brain was much lower compared to the titers in other organs. Taken together, Lassa virus is pantropic in IFNAR^-/-^ mice. The organ titers are consistent with the level of viremia and markers of disease for the different viruses.

### Histopathology of arenavirus infection in IFNAR^-/-^ mice

**Figure 3:**
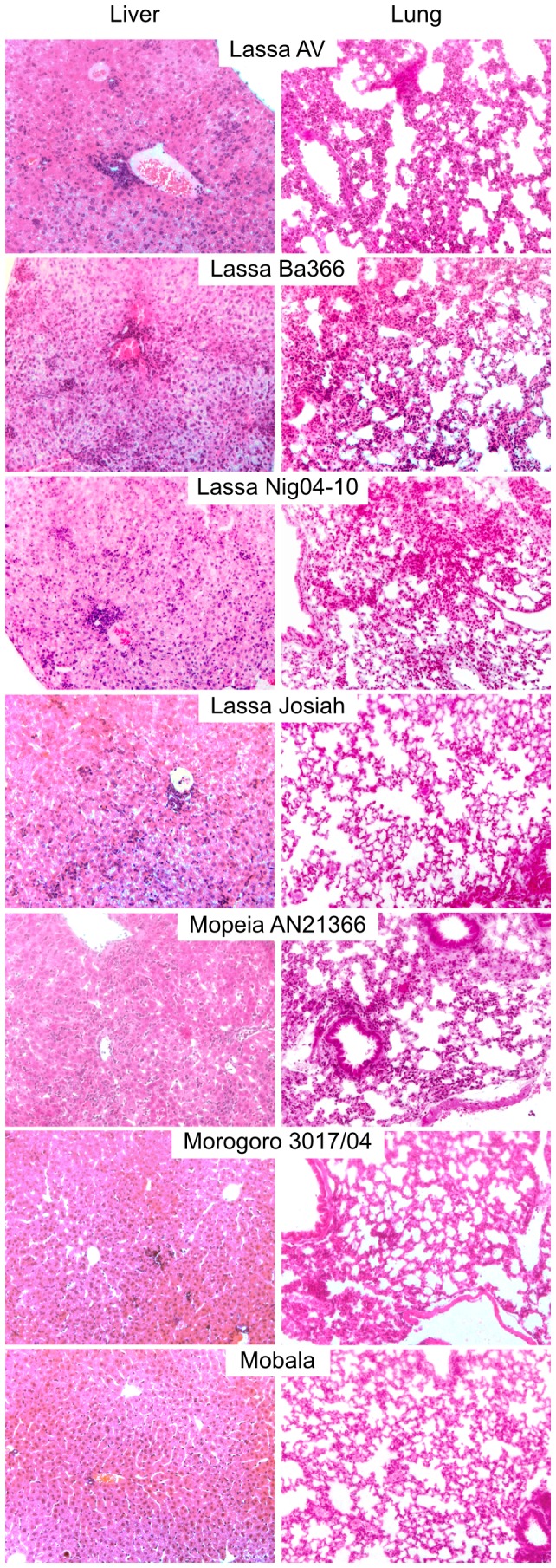
H/E-stained sections of liver and lung of infected IFNAR^-/-^ mice. Animals were inoculated i.v. with 10^3^ FFU of the indicated viruses and euthanized at day 9–10 p.i. to collect the organs.

**Figure 4:**
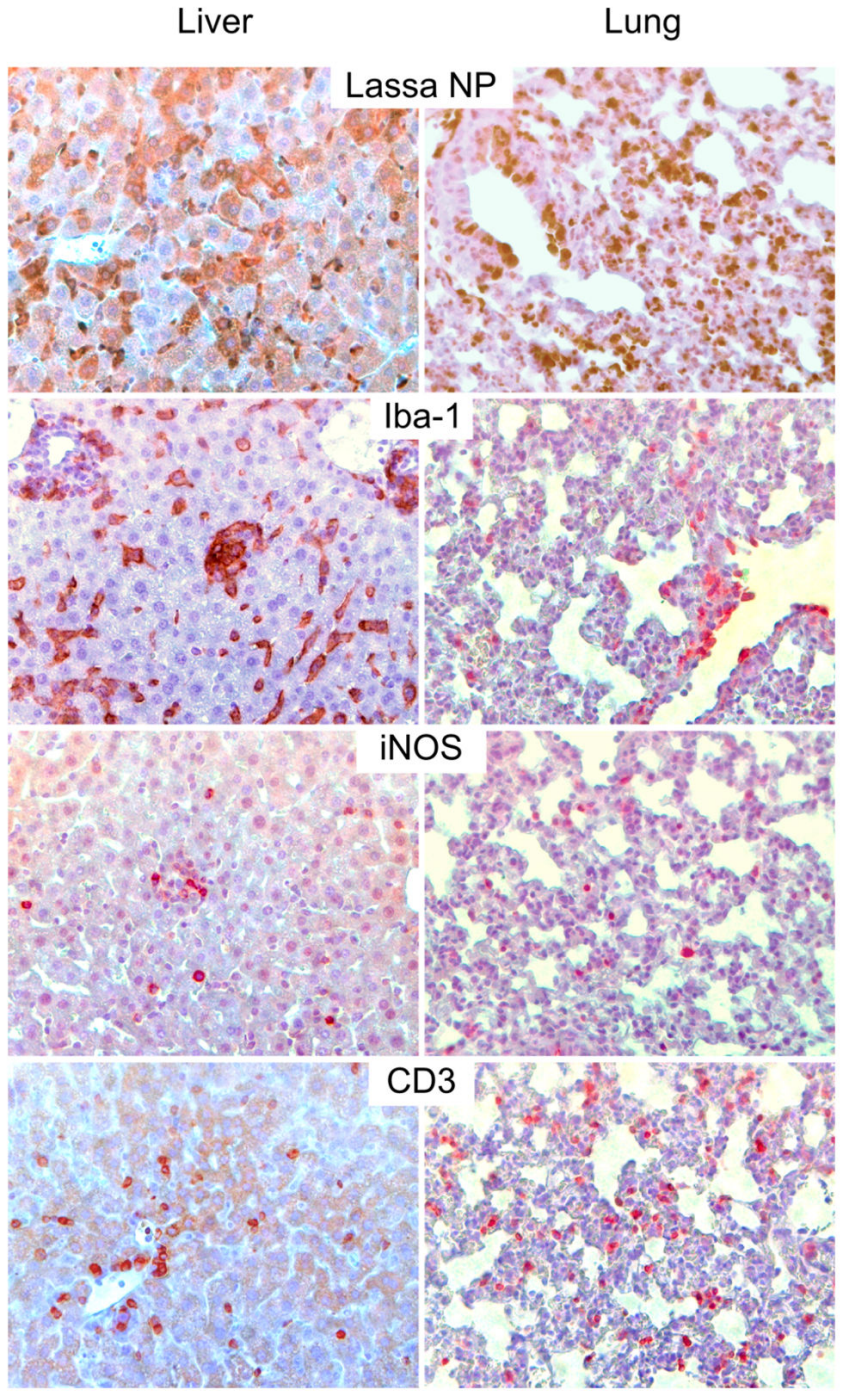
Immunohistochemistry of liver and lung of infected IFNAR^-/-^ mice. Animals were inoculated i.v. with 10^3^ FFU of Lassa virus strain AV and euthanized at day 9 p.i. to collect the organs. Sections were stained with anti-Lassa virus NP, anti-Iba-1, anti-iNOS, or anti-CD3 antibodies.

Collected organs (lung, kidney, heart, spleen, brain, and liver) of infected mice were assessed on H/E-stained sections and viral distribution in situ was assessed by immunostaining for NP at days 9–10 p.i. Pathological findings were largely identical for the Lassa virus strains and the apathogenic viruses, respectively. Therefore, they are described collectively for each group. **Liver**: Infection with Lassa virus was associated with acute hepatitis, as evidenced by periportal mononuclear inflammatory cell infiltrate disrupting the limiting plate of hepatocytes, and focal necrosis (Figure 3). The architecture of Iba-1-positive hepatic monocytes/macrophages (Kupffer cells) was disturbed (Figure 4). The cells were enlarged, rounded up, disorganized, and often accumulated in clusters (under physiological conditions, these cells form a flat layer along liver sinusoids). In addition, iNOS-expressing monocytes/macrophages, which are not present in normal liver, were detectable (Figure 4). These alterations are suggestive for monocyte/macrophage activation. T cells were scattered at moderate density throughout the liver parenchyma and in periportal inflammatory nodules. Lassa virus antigen was readily detected in a large fraction of hepatocytes and Kupffer cells (Figure 4). In contrast, Mobala, Mopeia, and Morogoro virus-infected animals were largely devoid of inflammatory infiltrates nor was viral antigen detectable (Figure 3). **Lung**: Lung tissue showed variable degree of interstitial pneumonia in Lassa virus-infected mice, but not in Mopeia, Morogoro, and Mobala virus-infected animals (Figure 3). Similar to the liver, the lung of Lassa virus-infected animals contained dense infiltrations of Iba-1-positive monocyte/macrophage lineage cells, iNOS-expressing monocytes/macrophages and accumulations of T cells (Figure 4). A large fraction of cells expressed Lassa virus antigen, consistent with the high virus titer in lung tissue. **Spleen**: Mice infected with Lassa virus showed pronounced alterations of follicular cytoarchitecture as evidenced by disturbed segregation of white and red pulpa. Immunostaining revealed widespread and numerous NP-positive cells in the parenchyma. In contrast, mice infected with Mobala, Mopeia, or Morogoro virus had a largely preserved follicular cytoarchitecture and infected cells were sparse or absent. **Kidney, heart, and brain**: In Lassa virus-infected mice, viral antigen could be detected in the endothelial cells of vasa recta (straight arterioles), in some glomeruli, and some interstitial cells of the kidney. Lassa virus antigen was sparsely present in the heart tissue with only few and focal spots of NP-positive endothelial cells, few macrophages, and isolated myocardiocytes. There were no signs of endo- or myocarditis. Brain samples showed focal Lassa virus NP-positive cells restricted to the brain coverings (ependymal cells and meninges) and some perivascular spots (most likely within astrocytes). In these perivascular areas, some activated microglial cells and isolated microglial nodules were noted that were associated with sparse lymphocytic infiltrates. Kidney, heart, and brain of Mobala, Mopeia, and Morogoro-virus infected mice showed no pathology and viral antigen was absent.

Taken together, mice infected with Lassa virus showed large numbers of infected cells in spleen, lung, and liver and to a lesser extent in kidney, heart, and brain. Main pathological alterations included acute hepatitis, pronounced disturbance of splenic cytoarchitecture, and signs of monocyte/macrophage activation. In contrast, in Mopeia, Morogoro and Mobala virus-infected animals only sparse viral antigen was detected in spleen and acute inflammatory alterations were not noted.

### Depletion of T cells in IFNAR^-/-^ mice infected with Mopeia, Morogoro, and Mobala virus

**Figure 5:**
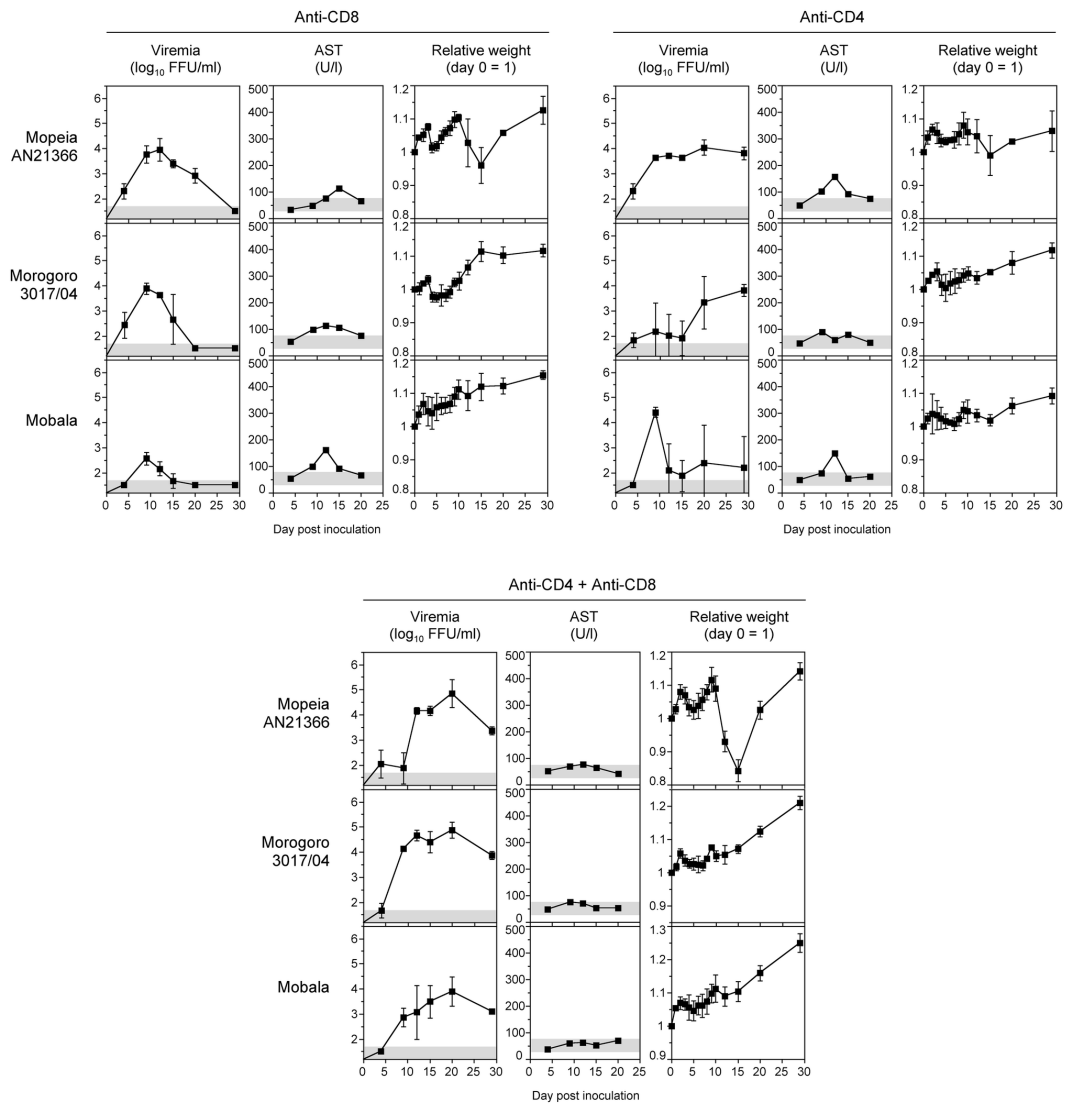
Influence of CD4^+^ and CD8^+^ T cells on susceptibility of IFNAR^-/-^ mice to apathogenic arenaviruses. CD8^+^ and/or CD4^+^ T cell populations were depleted by intraperitoneal administration of anti-CD8 and/or anti-CD4 on day -3 and day -1 of infection. Groups of three animals were inoculated i.v. with 10^3^ FFU of the indicated viruses. Mean and standard deviation are shown (n = 3). For AST measurement, blood of the three animals was pooled. The range of viremia below the detection limit of the immunofocusassay as well as the normal reference range of AST in mice [43] are shaded in grey.

The above experiments demonstrated that Mopeia, Morogoro, and Mobala virus hardly replicate in mice despite the absence of a functional type I interferon system, while Lassa virus is able to grow to high titers. Therefore, we wondered whether T cells might play a role in restricting the growth of the apathogenic viruses. To test this, IFNAR^-/-^ mice were depleted of CD8^+^ T cells or of CD4^+^ T cells or were depleted of both cell populations using anti-CD4 and anti-CD8 antibodies. Subsequently, the animals were inoculated with 10^3^ FFU Mopeia, Morogoro, or Mobala virus and observed for 30 days. Depletion of CD8^+^ T cells led to higher viremia of Mopeia and Morogoro virus, while no major change was seen for Mobala virus (Figure 5, top left). Virus was cleared during the observation period, though with slightly delayed kinetics compared to non-depleted mice. Depletion of CD4^+^ T cells led to enhanced replication of all three viruses (Figure 5, top right). However, the growth kinetics for Mopeia and Morogoro virus was slower than in non-depleted mice and both viruses were not cleared; the titer at the end of the 30-days observation period was still around 4 log_10_ FFU/ml. Similarly, one of three mice infected with Mobala virus did not clear the virus. Combined depletion of CD4^+^ and CD8^+^ T cells clearly enhanced growth of all three viruses with peak viremia of 4–5 log_10_ FFU/ml around day 20 (Figure 5, bottom). Virus was not cleared and still ranged between 3 and 4 log_10_ FFU/ml at the end of the experiment. While some biochemical evidence of disease was observed after single depletion, the level of AST was not elevated after double depletion. Taken together, depletion of CD4^+^ and CD8^+^ T cells enhanced susceptibility of IFNAR^-/-^ mice to Mopeia, Morogoro, and Mobala virus infection.

## Discussion

This study indicates that Lassa virus establishes productive infection in IFNAR^-/-^ mice independent of the virus strain. Main pathological alterations include acute hepatitis, pronounced disturbance of splenic cytoarchitecture, and morphological correlates of monocyte/macrophage activation. In contrast, Lassa virus-related arenaviruses, which are supposedly apathogenic for humans, hardly replicate and induce disease in these animals. CD4+ and CD8+ T cells were found to play a major role in resistance of IFNAR^-/-^ mice to the apathogenic arenaviruses.

A key finding of the study is that the virulence of the African arenaviruses Lassa, Mopeia, Morogoro, and Mobala in IFNAR^-/-^ mice correlates with their virulence in humans and non-human primates [33]. Even the low level of Mopeia virus replication and the corresponding AST elevation in the mice is consistent with histopathological and virological evidence of low-level Mopeia virus replication in non-human primates [31,33]. Thus, in both animal models Mopeia virus shows features that place it somewhere between Lassa virus and Morogoro/Mobala viruses. We consider these analogies an indication that relevant aspects of arenavirus infection in humans and non-human primates are reproduced by IFNAR^-/-^ mice. Elevation of AST with a high AST/ALT ratio, pantropism (virus could be recovered from all organs), hepatitis, interstitial pneumonia, and hepatic macrophage response as observed in Lassa virus-infected IFNAR^-/-^ mice are also key features of human Lassa fever [18,19,23,24]. Both AST and ALT are concentrated in the liver. However, while ALT is specific for this organ, AST is also present at high level in the heart, skeletal muscle, kidneys, brain, and red blood cells [44]. Therefore, the high AST/ALT ratio may indicate extrahepatic cell damage, which is consistent with the pantropism of the virus. One difference between the mouse model and non-human primate models of Lassa fever is the somewhat higher virus concentration in the brain of the mice relative to liver and lung [25,26,32]. However, we found only minor histopathological changes and the mice did not show overt neurological signs. The involvement of the brain in human Lassa fever is still poorly understood. Patients in the terminal phase often develop encephalopathy with severe neurological symptoms such as coma and seizures [22]. In one case, Lassa virus was found in cerebrospinal fluid, but not in serum [45], suggesting a tropism of Lassa virus for the central nervous system of humans. Further studies both in the mouse model and in humans are warranted to clarify the pathomechanism of Lassa fever-associated encephalopathy.

An advantage of the mouse model is that a broad range of reagents is available to dissect host pathways involved in pathogenesis. In addition, the difference in virulence between pathogenic and non-pathogenic arenaviruses in the IFNAR^-/-^ model offers the opportunity to study and identify viral factors determining virulence, e.g. by testing recombinant chimeric viruses. The viability of Lassa/Mopeia reassortant viruses has been shown and components of the replication complex can be exchanged between both viruses in the context of a replicon system without loss of activity [46,47]. A larger number of Lassa virus isolates has been tested previously only in inbred guinea pigs (which presumably are slightly immunocompromized due to the inbreeding and therefore more susceptible to pathogens than outbred animals). Most of the isolates were non-lethal for strain 2 guinea pigs, while about half of them were lethal for strain 13 guinea pigs [30]. The lethality and viremia patterns largely correlated with the severity of human disease, with some exceptions. The viremia and disease patterns of the Lassa virus strains tested here in the mouse model were nearly identical, and the disease was non-lethal. Testing further isolates from well defined human cases may disclose whether the mouse model also reflects differences in the severity of human Lassa fever.

Arenaviruses replicate in a specific rodent host species in nature, called the natural reservoir. Lassa, Mopeia, and Morogoro viruses share the rodent host *M. natalensis*, while Mobala virus is associated with *Praomys* sp. [7–12]. It is generally assumed that arenaviruses are highly adapted to their host species and may not easily infect another rodent species. However, reconstruction of virus–host co-phylogenies suggested that host-switching might have happened during arenavirus evolution in Africa [13]. It is less likely that barriers on the cellular level play a major role, as arenaviruses are able to replicate in cells of various species in vitro [48] and we have confirmed this for mouse cells: all viruses used in this study produced infectious foci on murine fibrosarcoma L929 cells, somewhat less efficient than on Vero cells but without difference between pathogenic and apathogenic viruses (data not shown). Obvious barriers preventing replication of arenaviruses in non-host rodent species are the MHC class I-restricted T cell response — Lassa virus productively infects MHC class I, but not class II knockout mice [17] — and the type I interferon response, as demonstrated previously and here for Lassa virus in mice [35,36]. It is conceivable that the interferon antagonistic activity of the virus, which is conferred by NP [49,50], is adapted to the interferon system of the natural host species and less efficient in non-host species. Rather surprisingly, the inactivation of the type I interferon system was not sufficient to overcome resistance of mice to Mopeia, Morogoro, and Mobala viruses. The T cell depletion experiments indicate that CD4^+^ and CD8^+^ T cells play a key role in restricting replication of these viruses in mice. Specifically the lack of CD4^+^ T cells appears to promote persistent infection. It is not clear which function of the T cells is important and whether T cell-specific cytokines or chemokines are involved. On the other hand, the fact that Lassa virus readily overcomes the species barrier in IFNAR^-/-^ mice without depletion of T cells suggests that Lassa virus is able to suppress T cell functions or T cell activation in a way that functionally corresponds to depletion.

Arenaviruses are rodent-born and thus most likely treated differently by the immune system of rodents compared to non-human primates and humans. Therefore, any correlation between the rodent model and humans must be done with great caution. Nevertheless, the parallels between arenavirus infection in IFNAR^-/-^ mice and non-human primates and humans may suggest that the mice model reflects certain aspects of human Lassa fever. Transferring the observations from IFNAR^-/-^ mice to humans would imply that the high pathogenicity of Lassa virus in humans, compared to Mopeia, Morogoro, or Mobala virus, is not primarily related to differences in the interaction with the human type I interferon system. Rather, it may be associated with the ability of Lassa virus to effectively downregulate T cell activation or functions. This hypothesis is in agreement with in vitro studies showing that human dendritic cells infected with Lassa virus are not activated and poorly stimulate human T cells, while Mopeia virus-infected dendritic cells are activated and strongly stimulate CD8^+^ and CD4^+^ T cells [14–16]. Moreover, it was shown that T cells are stimulated by Mopeia virus even if type I interferon and the corresponding receptor have been neutralized, suggesting that type I interferon is not essential for the induction of T cell responses against Mopeia virus [14]. These in vitro findings match with our in vivo observations that, in spite of type I interferon deficiency, T cells need to be depleted to facilitate growth of Mopeia virus in mice. The consistency between these different experimental systems further underlines that the IFNAR^-/-^ mice model may provide relevant insights into arenavirus biology.
